# Surgical management of lower limb radiculopathy following acute singe-level osteoporotic vertebral fracture of lower lumbar spine in geriatric patient: a retrospective study

**DOI:** 10.1186/s12891-024-07314-3

**Published:** 2024-04-03

**Authors:** Yao Zhang, Yuzheng Lu, Wancheng Lin, Mingtao Yao, Jipeng Song, Lixiang Ding

**Affiliations:** Department of Spinal Surgery, Beijing Shijitan Hospital, Capital Medical University, No. 10, tieyi road, Yangfangdian, Haidian district, Beijing, 100038 People’s Republic of China

**Keywords:** Osteoporotic vertebral fracture, Inferior endplate fracture, Radiculopathy, Foraminal stenosis, Percutaneous kyphoplasty

## Abstract

**Background:**

Radiculopathy of the lower limb after acute osteoporotic vertebral fractures (OVFs) in the lower lumbar spine is uncommon in geriatric patients. Moreover, surgical intervention is generally recommended in patients who are irresponsive to conservative treatment. Determining an optimum surgical strategy is challenging considering the poor general condition of this population. Thus, herein, we established an algorithm for surgically managing this clinical scenario, hoping to provide a reference for making a surgical decision.

**Methods:**

We retrospectively studied patients who suffered from new-onset radiculopathy of the lower limb after acute single-level OVFs in the lower lumbar spine and eventually underwent surgical intervention at our department. Information on the demographics, bone quality, AO spine classification of the vertebral fracture, pre-existing degenerative changes, including foraminal stenosis and lumbar disc herniation, and surgical intervention type was collected. Additionally, clinical outcomes, including preoperative and postoperative visual analog scale (VAS) scores for back and leg pain, Oswestry disability index (ODI), and MacNab criterion for response to surgery, were evaluated.

**Results:**

From September 2019 to December 2021, a total of 22 patients with a mean age of 68.59 ± 9.74 years were analyzed. The most involved vertebra was L5 (54.5%), followed by L4 (27.3%) and L3 (18.2%). Among the 22 patients, 15 (68.2%) were diagnosed with the A1 type fracture of AO classification, and among them, 11 (73.3%) were characterized by the collapse of the inferior end plate (IEP). Three patients (13.6%) suffered from A2-type fractures, whereas four patients (18.2%) suffered from A3-type fractures. Pre-existing degenerative changes were observed in 12 patients (54.5%) of the patients. A total of 16 patients (72.7%) were treated by percutaneous kyphoplasty (PKP). Additionally, three patients underwent posterior instrumentation and fusion, two patients underwent a secondary endoscopic foraminoplasty, and one patient underwent a secondary radiofrequency ablation. The mean follow-up period was 17.42 ± 9.62 months. The mean VAS scores for leg and back pain and ODI decreased significantly after the surgery (*P* < 0.05). The total satisfaction rate at the last follow-up was 90.9% per the Macnab criterion.

**Conclusion:**

Patients with OVFs in the IEP are predisposed to suffer from radiculopathy of the lower limb. PKP alone or in combination with other minimally invasive surgical strategies is safe and effective in treating stable fractures. Additionally, aggressive surgical intervention should be considered in patients with unstable fractures or severe foraminal encroachment.

## Introduction

Osteoporotic vertebral fractures (OVFs) resulting from a small trauma frequently, and sometimes spontaneously, occur in elderly patients [1]. OVFs are characterized by varying degrees of local pain, especially axial back pain, surrounding the fracture position [2]. Some patients with OVFs in the lower lumbar spine complain of post-traumatic new-onset radiating pain in the lower limbs, which is similar to the symptoms of lumbar disc herniation (LDH) or foraminal/canal stenosis, which is induced by intervertebral foramen encroachment resulting from decreased foraminal height and migrated fractured fragment [3]. For patients in which conservative treatment does not improve these symptoms, percutaneous vertebroplasty (PVP) or kyphoplasty (PKP) has been proven safe in relieving pain with reliable mechanical stabilization and foraminal height recovery [4–6]. However, a PVP or PKP alone may be unsuitable for certain patients with severe pre-existing degeneration or unstable fractures. Moreover, only a few researchers have reported the application of the combination of endoscopic foraminoplasty and PVP or instrumentation and fusion to manage such complicated conditions [7, 8]. Additionally, surgical intervention in such patients is challenging.

Thus, in the present study, we reviewed patients with new-onset radiculopathy in the lower limb, followed by acute OVFs in the lower lumbar spine. The patients underwent different surgical interventions per their conditions. Based on the collected data, we aim to establish a reference algorithm to manage this condition.

## Materials and methods

The present retrospective study was approved by the Ethics Review Board of Beijing Shijitan Hospital of Capital Medical University. All experiments were performed in accordance with the 1964 Helsinki Declaration and its later amendments or comparable ethical standards. All patients provided written informed consent to participate in this study.

From September 2019 to December 2021, patients who were diagnosed with acute single-level OVFs and hospitalized at our spine center for surgical intervention were retrospectively analyzed. The inclusion criteria were as follows: (1) age ≥ 60 years old, (2) presented with new-onset radiculopathy in the lower limb, followed by acute OVFs in the lower lumbar vertebra (L3, 4, and 5), (3) complete image data including preoperative and postoperative roentgen-ray, computed tomography (CT), and magnetic resonance images (MRI), and (4) symptoms of radiculopathy were in accordance with the imageological and physical check-up results. The exclusion criteria were as follows: (1) incomplete information including medical records, images, and follow-up data, (2) a follow-up period of less than 12 months, (3) fractures caused by major trauma, and (4) evidence of an inflammatory, neoplasm, infectious, or mental disease.

Further, information on demographics, bone quality (normal, osteopenia, and osteoporosis based on the distributional T-score of L1 to L4 according to the American and European guidelines [9, 10]), AO spine classification and stability of vertebral fractures (A1 and A2 fractures were identified as stable fractures [11], whereas the A3 fracture with double-end plates presented with obvious comminuted vertebral body comminution and significant loss of vertebral height or lumbar lordosis was regarded as the unstable fracture [12]), and the pre-existing degenerative changes (including foraminal stenosis, central canal stenosis, and spondylolisthesis, et al.) were evaluated.

The surgical intervention type, duration of operation, intraoperative blood loss, and related complications were recorded. Clinical outcomes were assessed before the surgery, after 1 week, 3 months, 6 months, 1 year of the surgery, and at the last follow-up. Visual analog scale (VAS) scores were calculated to analyze back and leg pain, whereas the Oswestry disability index (ODI) was calculated to evaluate functional capacity of patient with low back pain. The MacNab criterion was used at each follow-up to evaluate the opinions of the patients regarding treatment satisfaction.

### Statistical analysis

All data were statistically analyzed using SPSS (version 25, SPSS Inc, Chicago, Illinois). Continuous variables were described in the form of means ± standard deviations, whereas categorical variables were described in the form of frequencies and percentages. Student’s t-test for paired samples was used to compare the clinical outcomes VAS scores and ODI at each follow-up time point. Values at *P* < 0.05 were considered statistically significant.

## Results

Among the 624 patients reviewed, 22 patients (3.53%), which included 14 females, met the inclusion criteria and were enrolled in the present study. Their mean age was 68.59 ± 9.74 years (61–87 years), and the mean follow-up period was 17.42 ± 9.62 months (12–26 months). All patients underwent anti-osteoporosis therapy after the surgery for at least one year (mean duration: 14.96 ± 2.33 months). The most commonly involved lumbar vertebra was L5 (12 patients, 54.5%), and the A1-type fracture was the most common (15 patients, 68.2%). Among these 15 patients, 11 (80%) were characterized by the collapse of the inferior end plate (IEP). Two patients with the A3-type fracture presented the Kummel disease in the fractured vertebra. Additionally, pre-existing degeneration was observed in 12 patients (54.5%), and foraminal stenosis was the most common degeneration (7 patients, 31.8%). The detailed demographic and baseline characteristics of the patients are presented in Table 1.

**Table 1:** Demographics and baseline characteristics (*N* = 22)

Variables	Outcomes
**Age (mean ± SD) (years) (range)**	68.59 ± 9.74 (61–87)
**Gender (n) (%)**	
Male	8 (36.4%)
Female	14 (63.6%)
**BMI (mean ± SD) (range)**	24.66 ± 3.52; 21–30
**Smoking history**	9 (40.9%)
**Bone quality**^**a**^	
Normal	2 (9.1%)
Osteopenia	3 (13.6%)
Osteoporosis	17 (77.3%)
**Chronic comorbidity (n) (%)**	
Hypertension	6 (27.3%)
diabetes mellitus	4 (18.2%)
cerebral infarction	2 (9.1%)
cardiac insufficiency	3 (13.6%)
COPD	1 (4.5%)
Asthma	1 (4.5%)
**Fractured segment (n) (%)**	
L3	4 (18.2%)
L4	6 (27.3%)
L5	12 (54.5%)
**Type of fracture (n) (%)**	
A1	15 (68.2%)
A2	3 (13.6%)
A3	4 (18.2%)
**Fracture characteristics**^**b**^**(n) (%)**	
SEP	6 (27.3%)
IEP	11 (50.0%)
SEP + IEP	5 (22.7%)
canal encroachment	4 (18.2%)
Kummel disease	2 (9.1%)
**Preexisting degeneration (n) (%)**	
LDH	2 (9.1%)
foraminal stenosis	7 (31.8%)
canal stenosis	3 (13.6%)
Total	12 (54.5%)
**Anti-osteoporotic therapy (n) (%)**	
Denosumab	15 (68.2%)
Zoledronic Acid	7 (31.8%)
Duration (mean ± SD) (month)	14.96 ± 2.33

*COPD* Chronic obstructive pulmonary disease, *SEP* Superior end-plate, *IEP* Inferior end-plate, *LDH* Lumbar disc herniation, *SD* Standard deviation

^a^the bone quality was evaluated by the distributional T-score

^b^some patients showed coexisting fracture characteristics

Regarding surgical intervention types, 16 patients (72.8%) underwent PKP alone, 2 patients (9.1%, patient NO. 11 and 16) underwent posterior instrumentation and posterolateral fusion, 1 patient (4.5%, patient NO. 7) underwent posterior instrumentation and intra-vertebra allograft, 2 patients (9.1%, patient NO. 5 and 21) underwent PKP and endoscopic foraminoplasty, and 1 patient (4.5%, patient NO. 22) underwent PKP and radiofrequency ablation.

Regarding surgery-related complications, asymptomatic bone cement leakage was observed in six patients (27.3%) who underwent a PKP alone and were characterized by fractures in both end plates. Cerebrospinal fluid (CSF) leakage occurred in one patient (4.5%, patient NO. 16) with the unstable A3-type fracture who underwent posterior instrumentation and fusion. Two patients (9.1%, patient NO. 7 and 16) suffered from wound infection after the fusion surgery, and the infection was alleviated after antibiotic treatment for 2 weeks and daily drape changing. The diagnose and therapeutic characteristics of all patients are listed in Table 2. No revision surgery was needed and no delay-onset complications were observed in these patients during the follow-up. The surgical characteristics of patients who underwent open surgery (patient NO. 7, 11, and 16) are shown in Table 3.

**Fig. 1:**
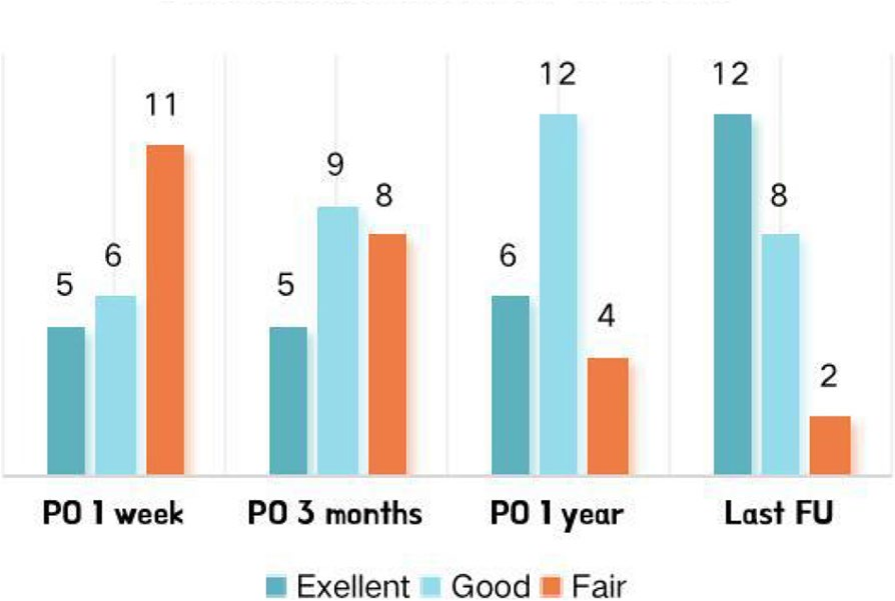
The patient’s opinion on treatment satisfaction at each follow-up (*N* = 22). PO indicates postoperative; FU indicates follow-up

Back pain in all patients improved significantly after the first surgery. Sixteen patients who underwent a PKP alone and three patients who underwent a fusion surgery alone showed significantly ameliorated radiculopathy during each postoperative follow-up. Furthermore, radiculopathy in the three patients (NO. 5, 21, and 22) improved significantly after the second surgery. VAS scores for back and leg pain decreased significantly from 7.33 ± 1.66 and 6.89 ± 0.76 (preoperative) to 2.61 ± 1.33 and 2.31 ± 0.92 (final) (*P* < 0.05), respectively. ODIs calculated preoperatively decreased significantly compared with those calculated during postoperative follow-ups (58.21 ± 10.38 vs. 19.32 ± 4.69, *P* < 0.05), as presented in Table 4. At the last follow-up, 20 patients (90.9%) showed satisfactory results according to the MacNab criteria,as illustrated in Fig. 1.

**Table 2:** Diagnose and therapeutic characteristics of the study patients

	Fracture level	Classification	Fracture characteristics	Preexisting degeneration	ASA	First surgery	Second surgery	Complication
NO. 1	L5	A1	IEP	N	II	PKP	N/A	N/A
NO. 2	L4	A1	IEP	foraminal stenosis	II	PKP	N/A	N/A
NO. 3	L5	A3	SEP	N	III	PKP	N/A	asymptomatic cement leakage
NO. 4	L5	A1	SEP	N	II	PKP	N/A	N/A
NO. 5	L4	A1	IEP	LDH	II	PKP	endoscopic disectomy	N/A
NO. 6	L4	A2	SEP + IEP	foraminal stenosis	II	PKP	N/A	asymptomatic cement leakage
NO. 7	L3	A3	SEP + IEP	foraminal stenosis	III	Posterior fusion	N/A	wound infection
NO. 8	L4	A1	IEP	N	III	PKP	N/A	N/A
NO. 9	L5	A1	SEP	foraminal stenosis	III	PKP	N/A	N/A
NO. 10	L5	A1	IEP	canal stenosis	II	PKP	N/A	asymptomatic cement leakage
NO. 11	L3	A2	SEP + IEP	canal stenosis	II	Posterior fusion	N/A	asymptomatic cement leakage
NO. 12	L5	A1	SEP	N	III	PKP	N/A	N/A
NO. 13	L3	A1	IEP	N	III	PKP	N/A	N/A
NO. 14	L5	A1	IEP	N	II	PKP	N/A	N/A
NO. 15	L5	A2	SEP + IEP	N	III	PKP	N/A	asymptomatic cement leakage
NO. 16	L5	A3	SEP + IEP	canal stenosis and de novo scoliosis	II	Posterior fusion	N/A	CSF leakage, wound infection
NO. 17	L5	A1	IEP	N	II	PKP	N/A	N/A
NO. 18	L4	A3	SEP	foraminal stenosis	II	PKP	N/A	asymptomatic cement leakage
NO. 19	L5	A1	IEP	N	III	PKP	N/A	N/A
NO. 20	L4	A1	IEP	foraminal stenosis	III	PKP	N/A	N/A
NO. 21	L5	A1	IEP	foraminal stenosis	II	PKP	endoscopic disectomy	N/A
NO. 22	L5	A1	SEP	LDH	III	PKP	radiofrequency ablation	N/A

*IEP* Inferior end plate, *SEP* Superior end plate, *ASA* American Standards Association, *CSF* cerebrospinal Fluid, *N* None, *N/A* Not applicable

**Table 3:** Surgical characteristics of the patients who underwent open surgery

	Blood loss (ml)	Duration of operation (min)	Instrumented segments	Fusion method
NO. 7	250	70	3	Intra-vertebral body allograft
NO. 11	330	90	3	Posterolateral autograft
NO. 16	300	90	3	Posterolateral autograft

**Table 4:** Comparison of clinical results between preoperative evaluation and each postoperative follow-up

	Pre-op	1-week	3-month	1-year	Final	P^1^	P^2^	P^3^	P^4^
**VAS**									
Back	7.33 ± 1.66	5.67 ± 2.13	4.78 ± 2.11	3.52 ± 1.34	2.61 ± 1.33	**0.0062**	**< 0.0001**	**< 0.0001**	**< 0.0001**
Leg	6.89 ± 0.76	4.01 ± 1.98	3.82 ± 1.33	3.02 ± 1.51	2.31 ± 0.92	**< 0.0001**	**< 0.0001**	**< 0.0001**	**< 0.0001**
**ODI**	58.21 ± 10.38	48.66 ± 12.69	35.62 ± 10.02	29.95 ± 5.46	19.32 ± 4.69	**0.0092**	**< 0.0001**	**< 0.0001**	**< 0.0001**

Pre-op: the preoperative follow-up; *P*^1^: comparison between the postoperative 1-week follow-up and the preoperative evaluations; *P*^2^: comparison between the postoperative 3-month follow-up and the preoperative evaluations;* P*^3^: comparison between the postoperative 1-year follow-up and the preoperative evaluations; *P*^4^: comparison between the last follow-up and the preoperative evaluations; the bold indicates significant difference

### Case presentation

#### Patient NO. 1: without pre-existing degeneration and treated by a PKP alone (Fig. 2)

A 68-year-old female experienced radiating calf pain in the right leg (L5 dermatome) and back pain for 10 days after an accidental fall. VAS scores for back and leg pain were 6 and 7, respectively. MRI revealed a fracture in the L5 IEP and decreased posterior height of the vertebra. No fractured fragment and herniated intervertebral disc infringing the foramen were observed. Thus, we performed PKP in which 10 mL of bone cement was used to restore the height of the vertebral body and foramen. The surgery significantly alleviated the back and leg pain, and the patient was satisfied with the treatment.

**Fig. 2:**
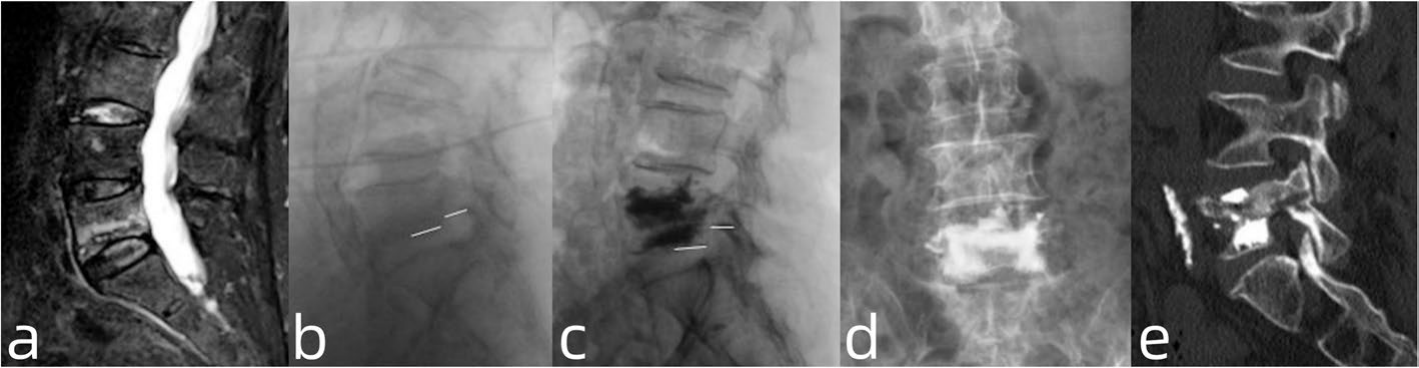
**a** Sagittal view of T2-weighted MRI showing the fracture of L5 IEP and decreased posterior vertebral body height; **b**, **c**: Comparison of pre- and post-operative radiography regarding the height between IEP and inferior margin of pedicle; **d**: Postoperative radiography showing satisfactory distribution of bone cements; **e**: The postoperative sagittal CT showing no bone cement leakage

#### Patient NO. 20: with pre-existing foraminal stenosis and treated by a PKP alone (Fig. 3)

An 80-year-old female suffered from radiating pain in the left leg (L4 dermatome) and back pain after falling from a stool. VAS scores for leg and back pain were 8 and 6, respectively. She was diagnosed with an A1-type OVF in the L4 vertebra, mainly the IEP, accompanied by L4/5 foraminal stenosis on the left side. Thus, we initially performed a PKP with the anticipation of cure for both the backache and radiculopathy owing to her poor general condition (ASA III). The VAS score for leg pain decreased to 4 immediately after the surgery. At the 3-month follow-up, the VAS score for leg pain was 3.

**Fig. 3:**
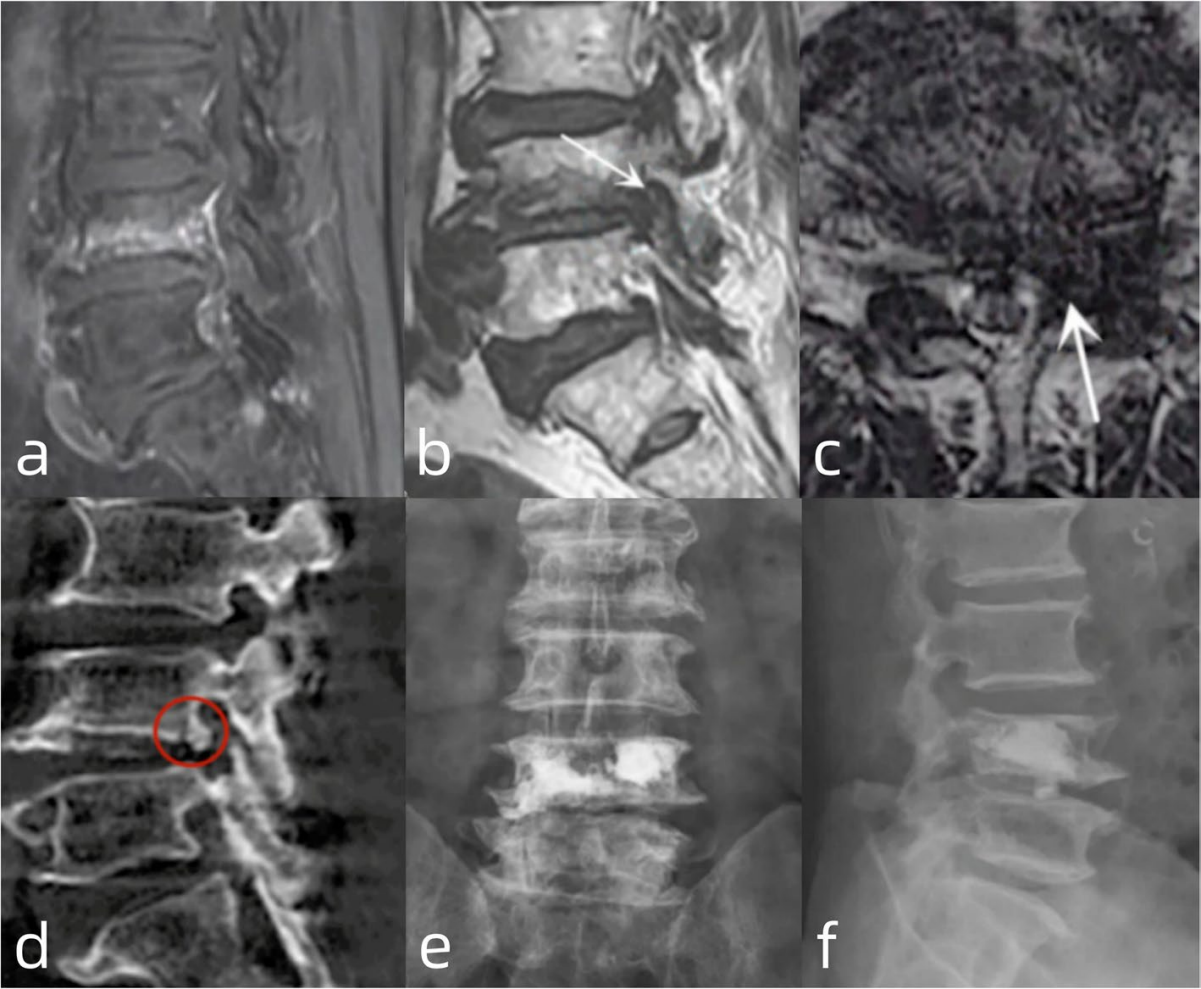
**a **Sagittal view of T2-weighted MRI showing fracture of L4 IEP; **b**, **c**: The sagittal and cross-sectional view of T2-weighted MRI identifying the stenosis of left-sided L4/5 foramen; **d**: Osteophyte and fractured fragment within the L4/5 foramen; **e**, **f**: The distribution of bone cement on anteroposterior and lateral view of postoperative radiography

#### Patient NO. 7: with pre-existing foraminal stenosis and treated by one-stage posterior short segmental instrumentation and fusion (Fig. 4)

A 66-year-old female visited our department for backache and radiating pain in the left leg after an accidental fall. Her VAS scores for back and leg pain were 8 and 6 points, respectively. Moreover, the physical examination showed an impaired left L3 nerve root, with decreased left patellar reflexes and quadricep muscle strength (grade 4). The preoperative radiography results revealed the significantly decreased height of the L3 vertebral body and lumbar lordosis. The CT and MRI of the lumbar spine confirmed an unstable A3-type fracture and L3 nerve root encroachment on the left. Additionally, the patient suffered from hypertension and type 2 diabetes mellitus, and the ASA was rated as grade II. Thus, we performed short-segmental fixation and transpedicular intra-vertebra allograft in combination with the over-extension posture and instrumentation. The intraoperative fluoroscopy showed the fractured vertebral height was satisfactorily reduced. An additional decompression procedure was not performed. The postoperative MRI showed satisfactory nerve root decompression. VAS scores for back and left leg pain at the postoperative 1-week follow-up were 5 and 4 points, respectively. The patient suffered from wound infection because of hyperglycemia, and the infection was cured after a 2-week antibiotic treatment and daily drape changing.

**Fig. 4:**
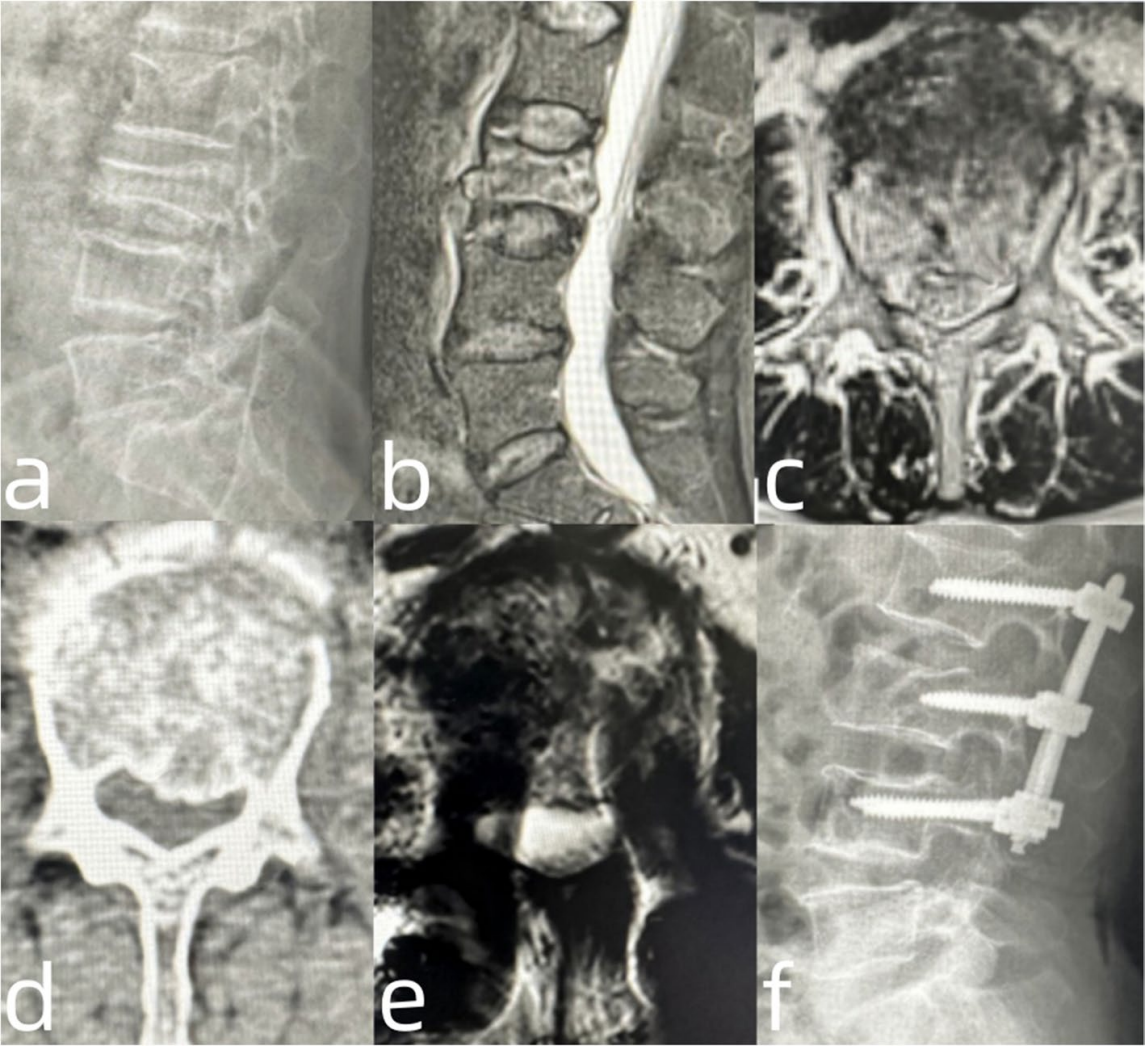
**a** The lateral view of the preoperative radiography of lumbar spine showing that the L3 vertebral height was significantly decreased and the lumbar lordosis was lost; **b**, **c**, **d**: Preoperative MRI and CT of lumbar spine manifesting a burst fracture of L3 vertebral body and encroachment of the central canal and left lateral recess on L3 vertebra; **e**: Postoperative cross-sectional MRI showing a well decompression of the central lateral and lateral recess; **f**: Postoperative lateral radiography showing a satisfactory reduction of L3 vertebral body height and an increase in lumbar lordosis

#### Patient NO. 21: with pre-existing foraminal stenosis and treated by PKP and endoscopic discectomy (Fig. 5)

A 75-year-old male presented to our department with the main complaint of pain in the lower back and right leg caused by lifting a wooden box. VAS scores for back and leg pain were 7 and 6 points, respectively. The physical examination revealed a decreased motion range in the lower lumbar spine and significant deep tenderness in the L4 spinous process. Furthermore, the sensation of the right lateral calf and patellar reflex decreased. Muscle strength in the right-sided quadriceps was of grade 4. Preoperative radiography and CT revealed an L4 A1-type fracture, which mainly involved the IEP. MRI showed that the right foramen was narrowed because of the pre-existing degenerative thickened ligamentum flavum and decreased vertebral height. Thus, we initially performed a PKP surgery. After the surgery, his backache improved significantly, and the VAS score was 3 points. However, pain in the right leg persisted, with a VAS score of 6 points. Thus, we performed endoscopic L3/4 foraminoplasty and the decompression of the L4 traversing nerve root. After the second surgery, the leg pain was relieved, and the VAS score was 3 points.

**Fig. 5:**
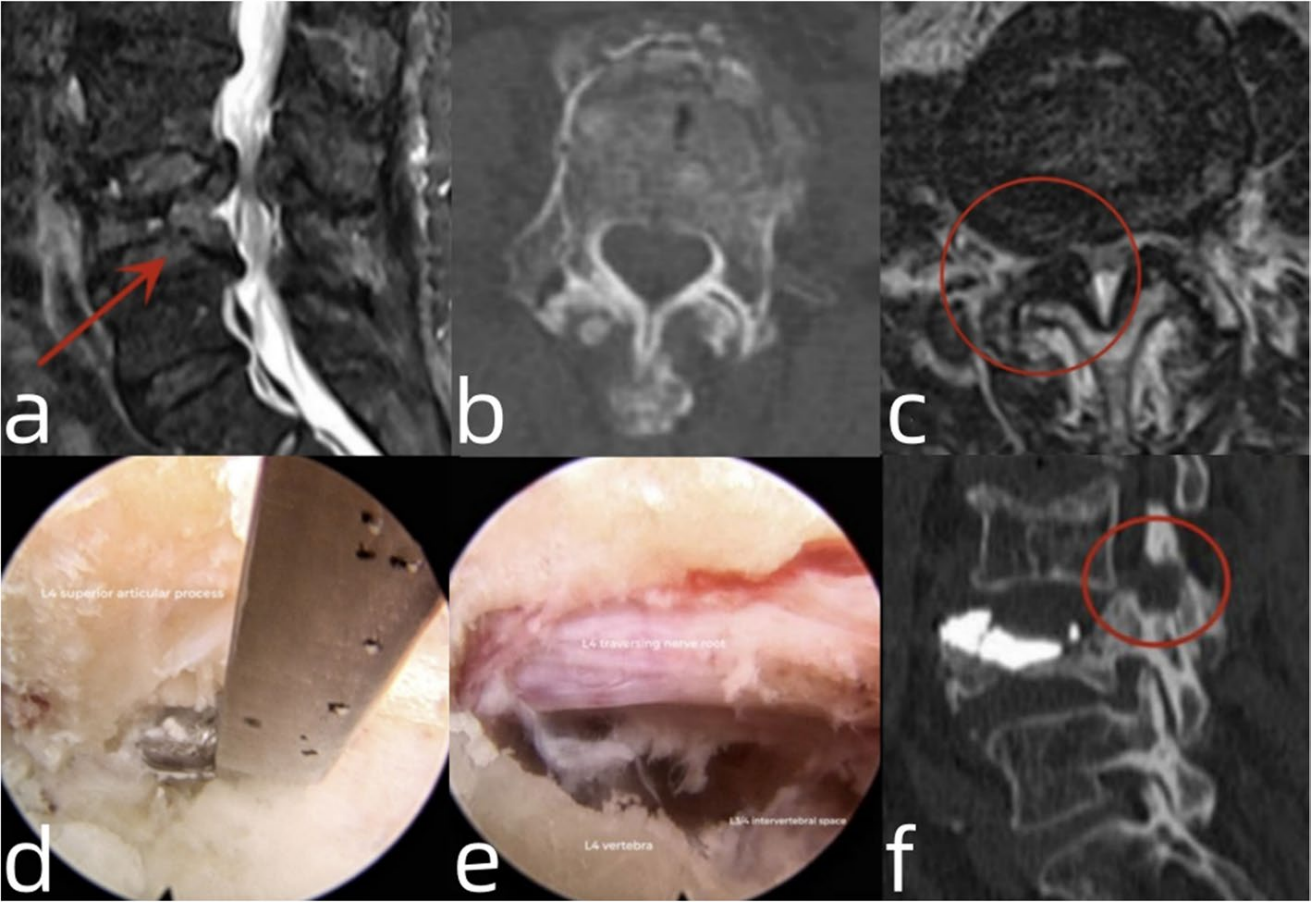
**a **Sagittal view of T2-weighted MRI revealing the fracture of L4 vertebra; **b**: Cross-sectional CT showing fracture of L4 IEP; **c**: Cross-sectional MRI showing stenosis of the right L3/4 foramen; **d**: Foraminoplasty on L3/4 with a Kerrison rongeur under full-endoscopic view; **e**: The L4 traversing nerve root was completely decompressed; **f**: Postoperative sagittal CT showing a partial resection of L4 superior articular process and pedicle, with no cement leakage in the foramen

## Discussion

With the aging of the population worldwide, OVF incidence is increasing [13]. Sometimes, OVFs do not manifest refractory back pain but result in neurological symptoms in the lower extremities. However, studies on such clinical conditions are lacking. The etiology of neurological impairment after an OVF in the lower lumbar spine can be attributed to the following: (1) migrated bony fragments invade the foraminal space, resulting in direct nerve root compression, (2) the decreased foraminal height after vertebral body collapse, mainly the posterior height, exacerbates the primary foraminal stenosis, and (3) posterior branches irritated by the narrowing foramen lead to pain in their distribution areas referred to as a radicular or somatic pain [14]. Because the IEP directly the anterior margin of the foramen, a vertebral fracture, which mainly involves the IEP, is susceptible to radiculopathy.

Ito et al. [15] reported that 55% of patients with OVFs exhibited neurological symptoms. However, patients with OVFs in the upper lumbar and thoracic spine were included in the study. Kim et al. [5] reported that the incidence of newly developed leg pain after the A1-type OVF was 25%, in which 86.7% of the patients suffered from IEP-type fractures in the lower lumbar spine. Conversely, in the present study, we observed that the incidence of radiculopathy of the lower limb after acute OVFs in the lower lumbar spine was 3.53% (22/624), in which the compressive fracture (A1 type) accounted for 68.2% of all fracture types. Moreover, patients with OVF involving IEP were more susceptible to radiculopathy of the lower extremities (72.7%) in comparison of those who characterized by SEP fracture (23.3%).

Herein, we observed 11 patients (50%) with pre-existing degeneration because degeneration frequently occurred in the lower lumbar spine. Based on pre-existing LDH or foraminal stenosis, radiculopathy symptoms after OVF occurrence worsened, indicating the need for a more aggressive intervention. In such clinical conditions in the geriatric population, surgical intervention is challenging because an open decompression with instrumented stabilization harbors a high risk of major surgery-related complications and fixation failure because of poor and reduced bone quality due to advanced age [16]. Kim et al. [5] successfully treated 15 elderly patients who suffered from radiculopathy after osteoporotic vertebral compression fractures by performing PKP/PVP alone. However, they only included the stable vertebral fractures (A1 type). Additionally, the patients they enrolled did not manifested with significant pre-existing lumbar degenerative changes. Zhao et al. [7] reported the use of PKP and combined full-endoscopic surgery in managing an elderly patient with pre-existing foraminal stenosis and radiculopathy after OVFs. Satisfactory clinical outcomes were observed in the patient at the 3-month follow-up. Lin et al. [17] reported 15 cases of radiculopathy after OVFs, which were treated with PKP/PVP and full endoscopic surgery. This treatment strategy improved back pain and neurological symptoms during the one-year follow-up. Herein, two patients with pre-existing LDH/foraminal stenosis were managed by performing PKP and full-endoscopic surgery, whereas one patient with LDH having poor general conditions underwent radiofrequency ablation after the first PKP. During the follow-up, satisfactory outcomes with no recurrence were observed in these patients.

Minimally invasive surgical interventions can be the most suitable option for elderly patients with poor general conditions and multiple comorbidities. Nonetheless, unstable fractures can occur frequently in the osteoporotic population even after a minor trauma [18]. Lee et al. [19] reported four elderly patients with unstable AO type III fractures who suffered from delayed-onset radiculopathy caused by refractures in the primarily operated vertebral body and retropulsed bone fragment 2–16 weeks after the initial PKP surgery. Next, these patients underwent posterior decompression and instrumentation, in which one patient died of postoperative intracranial hemorrhage. The refracture rate of the cemented vertebrae after PVP was as high as 63% in patients with osteoporosis [20], especially in those cases who had an over-restoration of the anterior height of vertebral body [21]. An adequate restoration of the posterior height of vertebral body is important to decrease the refracture rate of the cemented vertebra [21]. Revision surgery should be avoided as much as possible considering the poor general conditions of such elderly patients. Herein, we managed the most unstable A3-type fractures by performing one-stage open surgery, in which three patients with the fracture characterized by comminuted vertebra and loss of vertebral height and lumbar lordosis underwent short-segmental fixation and fusion. Moreover, surgical trauma can be minimized by not performing decompression if satisfactory reduction can be achieved after postural reduction or fixation, similar to that achieved in patient NO. 7. Based on the available follow-up data of the present study, the rate of surgery-related complications, including CSF leakage and wound infection, was 9.1%. All patients with complications showed a complete recovery after careful management. No delay-onset complications were observed.

**Fig. 6:**
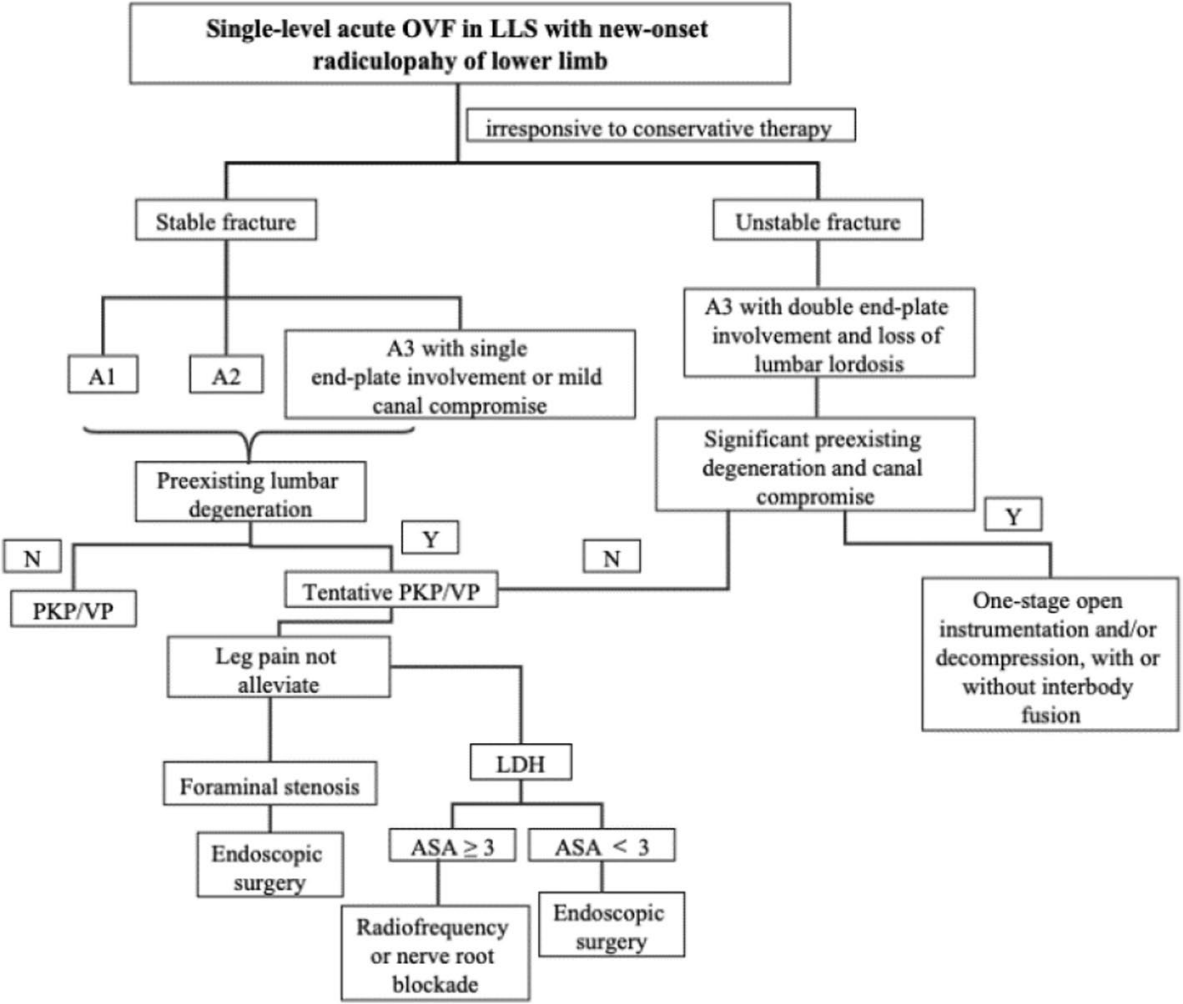
Flow chart depicting the surgical strategy for radiculopathy following acute OVF in the lower lumbar spine

Based on the present results, we provide a flow chart that will help decide an optimal surgical strategy to manage radiculopathy after acute OVFs in the lower lumbar spine, as illustrated in Fig. 6.

Nevertheless, the present study has several limitations. First, we excluded patients with a follow-up period of less than 12 months; thus, only 22 patients (3.53%) were included. This small sample size may not reflect the actual prevalence of radiculopathy after acute OVFs. Thus, studies with larger sample sizes should be performed to further estimate the actual incidence of new-onset radiculopathy after acute OVFs and to validate the present results. Second, the follow-up period was short with an average of 17.42 ± 9.62 months. However, each enrolled patient underwent a follow-up of at least one year. Third, we did not evaluate the imagological information at the final follow-up because of data loss, and evaluating this type of data is crucial to further evaluate long-term clinical outcomes. To address these limitations, randomized clinical trials for comparative studies in a larger population for a longer period should be conducted.

## Conclusion

Elderly patients with OVFs in the lower lumbar region characterized by IEP fractures and decreased posterior vertebral body heights are susceptible to radiculopathy of the lower limb. Selecting an appropriate surgical intervention strategy is critical to achieve satisfactory clinical outcomes. PKP aiming to restore the posterior vertebral and foraminal heights is an effective therapeutic strategy for patients without obvious degenerative changes. Additionally, endoscopic surgery or radiofrequency ablation can be a safe and effective supplement if patients do not respond to PKP. Short-segment fixation and fusion should be considered in patients with unstable A3-type fractures or severe canal encroachment.

## Data Availability

The data analyzed during the current study are available from the corresponding author upon reasonable request.
